# getENRICH: a tool for the gene and pathway enrichment analysis of non-model organisms

**DOI:** 10.1093/bioadv/vbaf023

**Published:** 2025-02-07

**Authors:** Ajay Bhatia, Pranjal Pruthi, Isha Chakraborty, Nityendra Shukla, Jitendra Narayan

**Affiliations:** CSIR-Institute of Genomics and Integrative Biology, New Delhi 110007, India; Academy of Scientific and Innovative Research (AcSIR), Ghaziabad 201002, Uttar Pradesh, India; CSIR-Institute of Genomics and Integrative Biology, New Delhi 110007, India; CSIR-Institute of Genomics and Integrative Biology, New Delhi 110007, India; CSIR-Institute of Genomics and Integrative Biology, New Delhi 110007, India; CSIR-Institute of Genomics and Integrative Biology, New Delhi 110007, India

## Abstract

**Motivation:**

The Gene Ontology system facilitates the functional annotation of genes by categorizing them into specific biological processes, cellular components, and molecular functions. Despite numerous tools like DAVID and Enrichr, analysing non-model organisms remains challenging due to a lack of genetic information and available tools.

**Results:**

To address this, we present getENRICH, a comprehensive tool for gene enrichment analysis tailored for non-model organisms. Available in both command-line and web-based graphical user interface (GUI) formats, getENRICH facilitates user-friendly interaction for gene dataset uploads, parameter configuration, and visualization. getENRICH employs hypergeometric distribution for *P*-value calculation and Benjamini–Hochberg correction for multiple testing.

**Availability and implementation:**

getENRICH is freely available under the MIT license, with the source code, documentation, and example datasets available on GitHub (https://github.com/jnarayan81/getENRICH) and the GUI version available at https://getenrich.igib.res.in/.

## 1 Introduction

The Gene Ontology (GO) system, constructed in 1998, created a framework for associating a set of genes to a biological function, thereby enabling researchers to link genes to a defined biological function (Ashburner *et al.* 2000). The advancement of high-throughput sequencing technologies has enabled biologists to quantify hundreds or even thousands of genes. Interpretation of such large datasets is exceptionally challenging, hence, the GO system allows for summarization of gene expression profiles into simplified functional categories, such as allowing for investigation of genome-wide changes and regulation of genes. Functional enrichment analysis, also known as gene-set enrichment analysis, is widely used in computational biology. It enables the assessment of gene sets for enrichment in specific pathways or biological functions, thereby leading to important functional insights into the discovery of novel biological functions or mechanisms. Alongside GO systems, the Kyoto Encyclopaedia of Genes and Genomes (KEGG) represents one of the comprehensive gene annotation databases. Numerous tools are available for high-throughput gene and pathway enrichment analysis, including, but not limited to DAVID (Sherman *et al.* 2022), Enrichr (Kuleshov *et al.* 2016), ShinyGO (Ge *et al.* 2020), and many more. These tools are largely accessible to researchers through graphical user-interface (GUI) and command-line interfaces (CLIs).

Despite the availability of various tools and databases for gene and pathway enrichment analysis, significant challenges persist, particularly for non-model organisms, some of which are widely studied, with notable examples being rotifers, avians, and tardigrades. Many existing resources and methodologies are optimized for model organisms with well-characterized genomes and extensive annotations. Consequently, researchers working with non-model organisms often encounter difficulties in obtaining accurate functional enrichment results due to limited or less comprehensive annotation data. To address these challenges, we have developed a new tool, getENRICH, to broaden the scope of enrichment analyses across a diverse array of organisms. This tool is specifically tailored to improve gene enrichment analysis for non-model organisms, providing enhanced capabilities to navigate the complexities associated with these less-characterized species. It offers both command-line and graphical user interface (GUI) options, facilitating the integration and interpretation of gene expression data across diverse biological contexts. By providing numerous features for visualizing enrichment results and ability to swiftly analyse large datasets, getENRICH provides a platform for researchers to extract meaningful biological insights from complex data.

## 2 Methods

**Figure 1.**
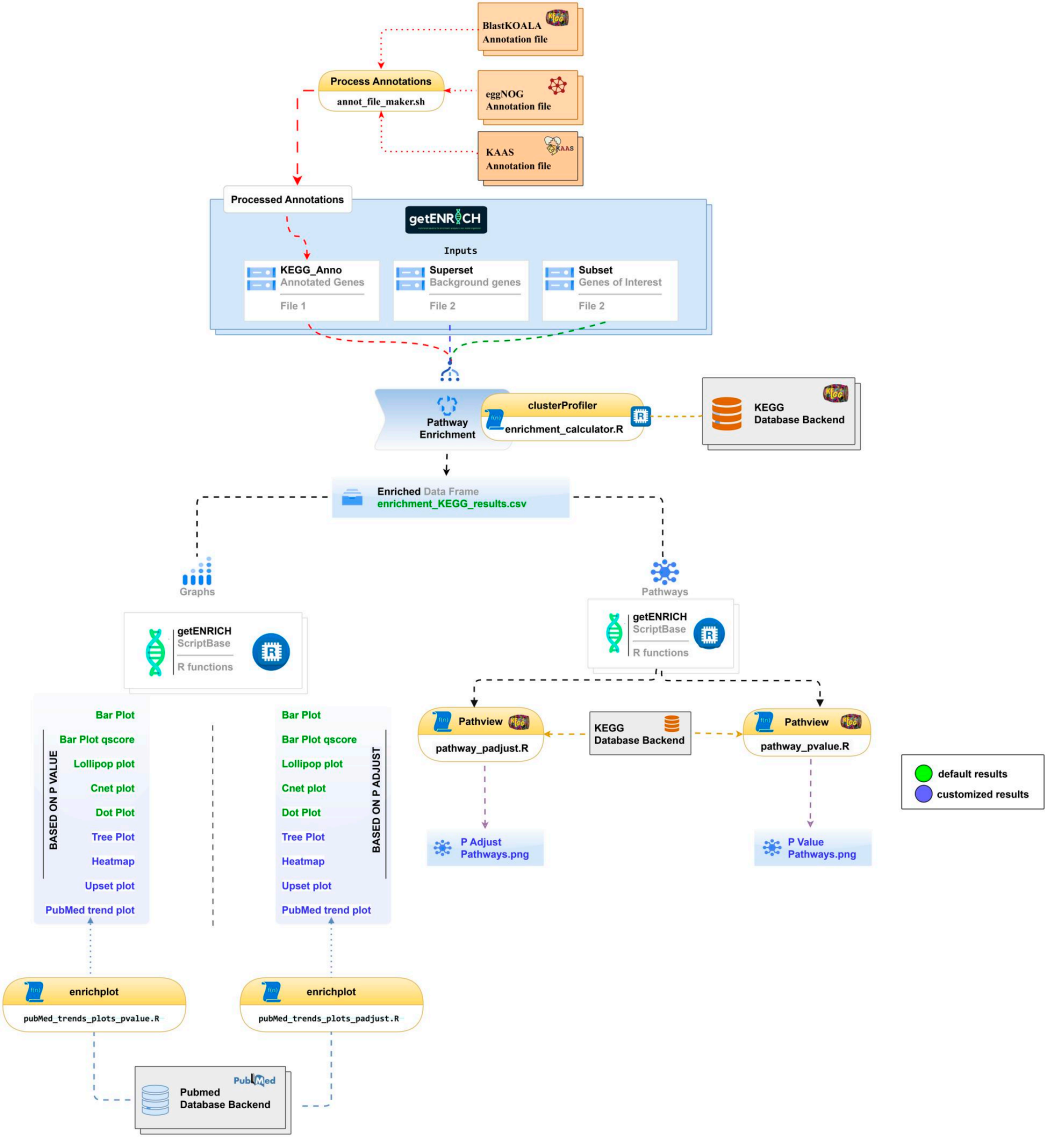
Detailed workflow of getENRICH packages and scripts used in the workflow, indicated using yellow and white boxes, respectively. In the graphs and pathways section, default and customised results are shown with green and blue colours, respectively.

getENRICH has been implemented in CLI by a combination of scripts in Shell and R, and thus requires very few dependencies to set up. It requires three input files, first being the KEGG Orthology (KO) annotation of the query genome, generated using tools like eggNOG-mapper (Cantalapiedra *et al.* 2021), BlastKOALA (Kanehisa *et al.* 2016), and KAAS (Moriya *et al.* 2007). The annotation files are processed for input in getENRICH using the annot_file_maker.sh, an in-house script that can take a maximum of three annotation files from the above-mentioned tools to create a sum of annotations or a minimum to process the data. The annotation file needed for input should be a tab-delimited file consisting of two columns, containing protein IDs and KO accession. The second and third input files are the background and foreground gene datasets, which should contain the protein IDs. Once the input is provided, the workflow (Fig. 1) proceeds and performs enrichment analysis, using the KEGG database to annotate gene pathways. *P*-value and *P*-adjusted value thresholds can be set according to the user’s needs and are set at a default value of 0.05. In addition, getENRICH creates various visualizations and pathway maps based on *P*-value and *P*-adjusted values that can be provided by the user through various flags. Visualizations include heatmaps, upset plots, treeplots, PubMed trend analysis plots, and significant pathway diagrams.

The GUI version of getENRICH, available at https://getenrich.igib.res.in/, also allows for swift customization and adjustment of various analysis parameters, such as the *P*-value and *P*-adjusted value thresholds, allowing for higher fine-tuning of statistical stringency, according to the user’s needs. Similar to the CLI, the user can opt to output their choice of visualizations. The web portal has a job submission process, allowing for users to submit multiple jobs at a time and track their progress in real time.

We utilized the clusterProfiler (Yu *et al.* 2012) package in R to calculate pathway enrichment statistically. We utilized the phyper function to perform hypergeometric distribution analysis. To control the false discovery rate during multiple tests, the adjusted *P*-values function is used with the method set to “BH” (Benjamini–Hochberg).

## 3 Results

**Figure 2.**
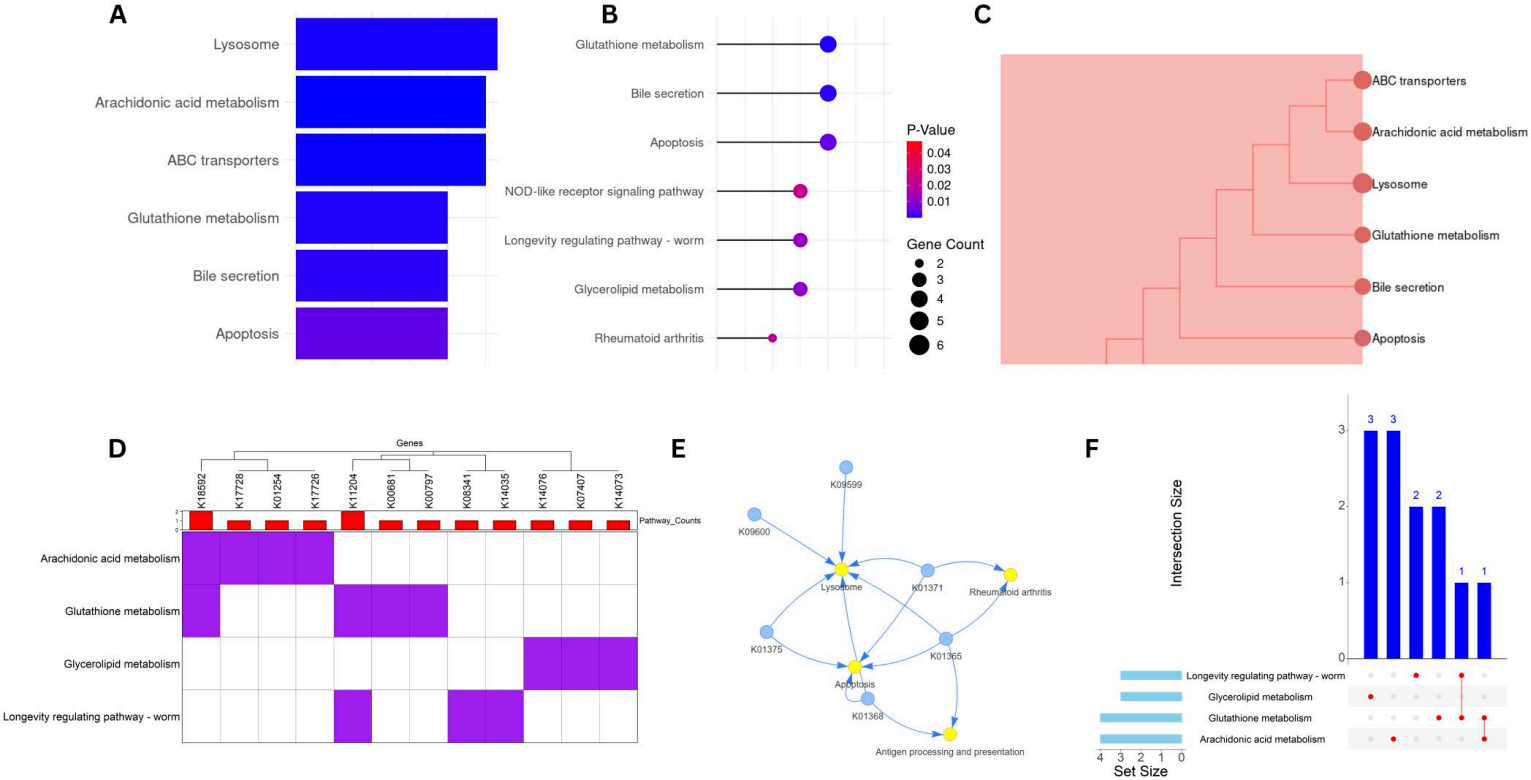
Example outputs of enrichment analysis of non-model organism *Adineta vaga* with getENRICH. (A) Barplot showcasing enrichment scores. (B) Lollipop plot. (C) TreePlot displaying hierarchical clustering of enriched terms. (D) Heatmap visualizing the distribution of genes of interest among different gene sets. (E) Cnetplot (pathway–gene network plot). (F) UpSet plot displaying the overlaps between various genes and pathways.

getENRICH was developed for comprehensive gene enrichment analysis of non-model organisms and provides comprehensive visualizations of enrichment, gene-pathway relationships, as well as publication trends (Fig. 2). Statistical tests, such as Fisher’s exact test and Benjamini–Hochberg method for multiple testing correction are performed to identify differences between query genes and background genes. We tested getENRICH on a publicly available genome assembly of *Adineta vaga* (GCA_021613535.1) (Simion *et al.* 2021). We selected 437 genes as the foreground dataset and 12 134 genes as the background dataset. We applied all the flags to generate all the graphs available, along with the KEGG pathway diagrams and kept the significance value at default (<0.05). As a result, we obtained all visualizations, according to significance scores of the *P*-value and the adjusted *P*-values value in both PNG and HTML format. Five and fifteen KEGG pathway diagrams were also included in the output, which were significantly enriched in adjusted *P*-values and *P*-value scores, respectively. A tabular output is also created, containing pathway information along with the *P*-value and adjusted *P*-values scores.

We compared getENRICH against other available enrichment analysis tools (Table 1). getENRICH holds the advantage over being available to both model and non-model organisms since different tools require a pre-existing protein annotation, often absent in non-model organisms, present in their query databases. getENRICH can accept KO annotations as input and perform ortholog-based enrichment analysis.

**Table 1.** Comparison of features offered by various popular enrichment analysis pipelines against getENRICH.

Parameter	Tools					
	getENRICH	ShinyGO	DAVID	Enrichr	PANTHER	g:Profiler
**Non-model organisms (all)**	Yes	No	No	No	No	No
**GUI**	Yes	Yes	Yes	Yes	Yes	Yes
**CLI**	Yes	No	No	No	No	No
**Data input format**	TXT, tab-delimited files	Text input	Text input, TXT, tab-delimited files	Text input, TXT, BED files	Text input, TXT, tab-delimited files	Text input, TXT, GMT files
**Interactive visualization options**	Yes	Yes	Yes	Yes	Yes	Yes
**Pathway diagrams**	Yes	Yes	Yes	No	Yes	No
**KEGG ortholog database**	Yes	Yes	Yes	Yes	No	Yes
**Fisher exact test**	Yes	Yes	Yes	Yes	Yes	Yes
**Benjamini–Hochberg procedure**	Yes	Yes	Yes	Yes	Yes	Yes

**Figure 3.**
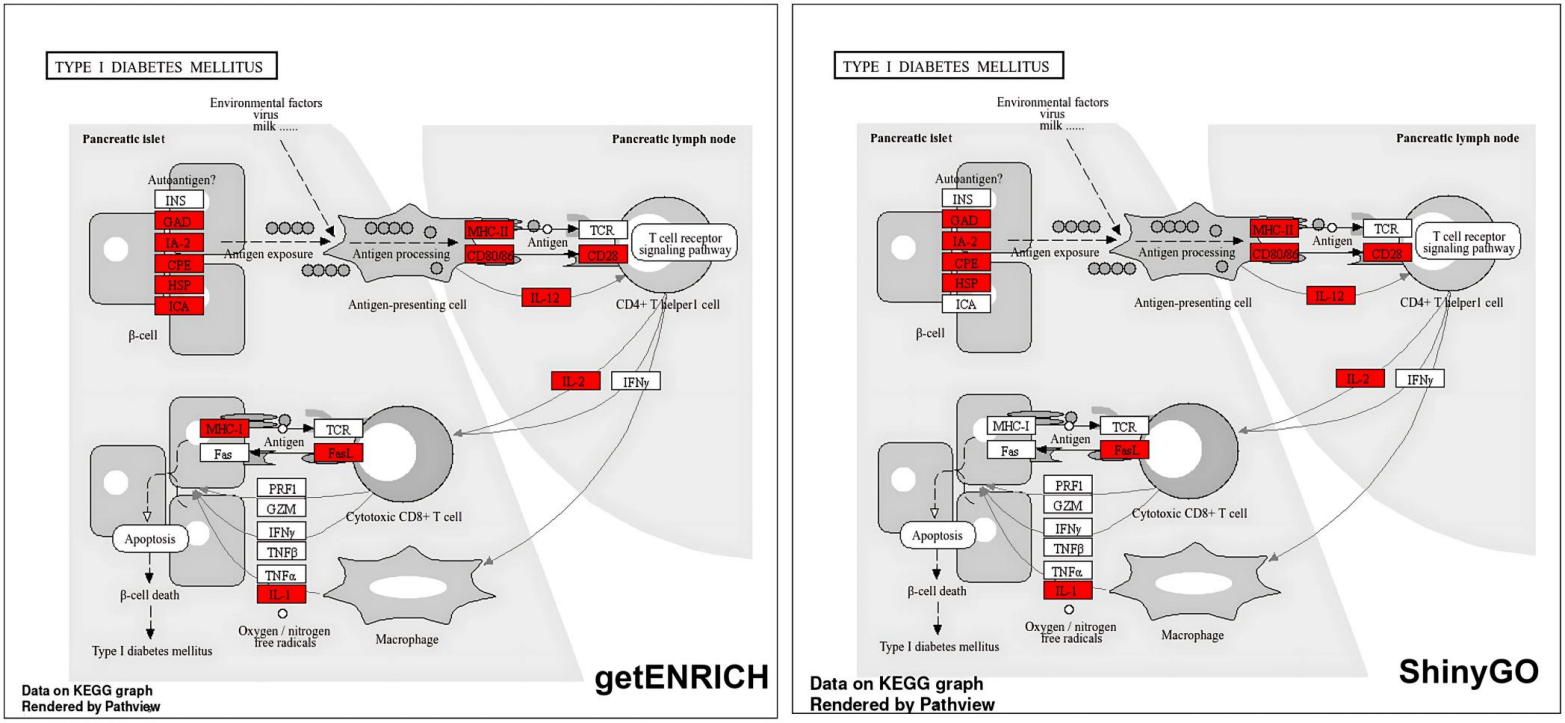
Gene enrichment comparison in Type-I Diabetes Mellitus between getENRICH and ShinyGO. Enriched genes are displayed in red. getENRICH had two additional genes (MHC-I and ICA) enriched compared to ShinyGO.

Additionally, we tested the functionality of getENRICH against model organisms, benchmarking it against ShinyGO, performing enrichment in the human genome. We retrieved the manually curated annotation for the human genome from the KEGG database through the clusterProfiler package. In order to maintain the consistency of inputs, we chose 13 046 genes as background and 3606 genes as foreground, sampled randomly, for our analysis. Since both background and foreground genes lists were the same for both the tools, all the enriched pathways overlapped across both pipelines, with 15 for getENRICH and 19 (Table 2) for ShinyGO being statistically significant (<0.05). As getENRICH is KO-based, additional genes may be enriched, since its analysis is based upon genes across different species that perform similar functions as opposed to gene-symbol based analysis that’s limited to the organism in ShinyGO. A notable example being the Type-I Diabetes Mellitus pathway (Fig. 3), where two additional genes, MHC-I and ICA, were enriched in our pipeline’s analysis but were absent in ShinyGO.

**Table 2.** List of statistically significant (*P* < .05) pathways sorted by adjusted *P*-values enriched by getENRICH and ShinyGO.

getENRICH					ShinyGO		
#	Pathway	Fold Enrichment	*P*-value	Adjusted *P*-value	#	FDR	Fold enrichment
1	Systemic lupus erythematosus	1.997526184	7.81E−11	2.69E-08	**4**	4.38E−10	2.153019275
2	Viral protein interaction with cytokine and cytokine receptor	1.840830273	2.32E−06	0.000277	**8**	5.49E−05	1.922406148
3	Neutrophil extracellular trap formation	1.570194712	2.42E−06	0.000277	**15**	5.49E−05	1.637193676
4	Drug metabolism—cytochrome P450	1.918926103	6.88E−06	0.000498	**6**	0.000172661	2.017339785
5	Ascorbate and aldarate metabolism	2.475651578	7.23E−06	0.000498	**1**	5.49E−05	2.759720826
6	Cytokine-cytokine receptor interaction	1.407212476	2.41E−05	0.00138	**17**	0.000241723	1.463530156
7	Hematopoietic cell lineage	1.717134271	5.57E−05	0.002564	**9**	0.000610858	1.815605807
8	Pentose and glucuronate interconversions	2.198769493	5.96E−05	0.002564	**2**	0.000936278	2.379069678
9	Metabolism of xenobiotics by cytochrome P450	1.684690992	0.000372	0.014211	**12**	0.006808958	1.762205636
10	Type I diabetes mellitus	2.16494227	0.000428	0.014738	**5**	0.028022194	2.118206775
11	Porphyrin metabolism	1.842778242	0.000886	0.027714	**7**	0.0158862	1.962817088
12	Legionellosis	1.724525093	0.001421	0.037612	**10**	0.0158862	1.815605807
13	Retinol metabolism	1.669574124	0.001421	0.037612	**11**	0.014402652	1.783753073
14	Biosynthesis of cofactors	1.402292152	0.001942	0.045086	**18**	0.026970084	1.432027115
15	Alcoholism	1.356112581	0.001966	0.045086	**19**	0.016071205	1.410985084
*	Allograft rejection				**3**	0.0158862	2.360287549
*	Drug metabolism-other enzymes				**13**	0.016071205	1.664305323
*	Chemical carcinogenesis-DNA adducts				**14**	0.037206212	1.639902019
*	Bile secretion				**16**	0.016071205	1.629389827

* Adjusted *P*-values were not significant in getENRICH but were significant in ShinyGO (*P* < 0.05).

## 4 Discussion

getENRICH allows for rapid, comprehensive KO-based enrichment analysis in non-model organisms, and has been made accessible on both CLI and GUI platforms for convenience, allowing users to tweak the algorithm according to their needs or perform quick enrichment analysis, with detailed outputs and visualizations providing crucial insights into gene set and pathway enrichment. No pipelines are without their limitations, and getENRICH is no exception. getENRICH relies on both the KEGG GENES database for KO-based annotation and KEGG database for enrichment analysis. Since we rely on a single database, some genes may not be annotated if a suitable ortholog is not found, which may affect the results. Additionally, pathway size can have a significant impact on results, and since KEGG has large pathway definitions, the sensitivity of the results may be affected. We will continue to improve getENRICH based on community recommendations as well as provide important updates and support.

## Data Availability

No new experimental data were generated for this study. Source code, example data, and documentation are publicly available at https://github.com/jnarayan81/getENRICH, with the web-version available at https://getenrich.igib.res.in/.
